# Multimodal and multiscale optical imaging of nanomedicine delivery across the blood-brain barrier upon sonopermeation

**DOI:** 10.7150/thno.41161

**Published:** 2020-01-12

**Authors:** Jan-Niklas May, Susanne K. Golombek, Maike Baues, Anshuman Dasgupta, Natascha Drude, Anne Rix, Dirk Rommel, Saskia von Stillfried, Lia Appold, Robert Pola, Michal Pechar, Louis van Bloois, Gert Storm, Alexander J. C. Kuehne, Felix Gremse, Benjamin Theek, Fabian Kiessling, Twan Lammers

**Affiliations:** 1 Institute for Experimental Molecular Imaging (ExMI), University Clinic and Helmholtz Institute for Biomedical Engineering, RWTH Aachen University, Aachen, Germany; 2 DWI - Leibniz Institute for Interactive Materials, RWTH Aachen University, Aachen, Germany; 3 Institute of Pathology, University Clinic RWTH Aachen, Aachen, Germany; 4 Czech Academy of Sciences, Institute of Macromolecular Chemistry, Prague, Czech Republic; 5 Department of Pharmaceutics, Utrecht Institute for Pharmaceutical Sciences, Utrecht University, Utrecht, The Netherlands; 6 Institute of Organic and Macromolecular Chemistry, Ulm University, Ulm, Germany; 7 Fraunhofer MEVIS, Institute for Medical Image Computing, Aachen, Germany; 8 Department of Targeted Therapeutics, University of Twente, Enschede, The Netherlands

**Keywords:** Ultrasound, Microbubbles, Nanomedicine, Drug delivery, Blood-brain barrier

## Abstract

**Rationale**: The blood-brain barrier (BBB) is a major obstacle for drug delivery to the brain. Sonopermeation, which relies on the combination of ultrasound and microbubbles, has emerged as a powerful tool to permeate the BBB, enabling the extravasation of drugs and drug delivery systems (DDS) to and into the central nervous system (CNS). When aiming to improve the treatment of high medical need brain disorders, it is important to systematically study nanomedicine translocation across the sonopermeated BBB. To this end, we here employed multimodal and multiscale optical imaging to investigate the impact of DDS size on brain accumulation, extravasation and penetration upon sonopermeation.

**Methods**: Two prototypic DDS, i.e. 10 nm-sized pHPMA polymers and 100 nm-sized PEGylated liposomes, were labeled with fluorophores and intravenously injected in healthy CD-1 nude mice. Upon sonopermeation, computed tomography-fluorescence molecular tomography, fluorescence reflectance imaging, fluorescence microscopy, confocal microscopy and stimulated emission depletion nanoscopy were used to study the effect of DDS size on their translocation across the BBB.

**Results**: Sonopermeation treatment enabled safe and efficient opening of the BBB, which was confirmed by staining extravasated endogenous IgG. No micro-hemorrhages, edema and necrosis were detected in H&E stainings. Multimodal and multiscale optical imaging showed that sonopermeation promoted the accumulation of nanocarriers in mouse brains, and that 10 nm-sized polymeric DDS accumulated more strongly and penetrated deeper into the brain than 100 nm-sized liposomes.

**Conclusions**: BBB opening via sonopermeation enables safe and efficient delivery of nanomedicine formulations to and into the brain. When looking at accumulation and penetration (and when neglecting issues such as drug loading capacity and therapeutic efficacy) smaller-sized DDS are found to be more suitable for drug delivery across the BBB than larger-sized DDS. These findings are valuable for better understanding and further developing nanomedicine-based strategies for the treatment of CNS disorders.

## Introduction

The blood-brain barrier (BBB) constitutes one of the most important barriers for successful drug treatment of neurological diseases. While permitting the passage of oxygen and nutrients, the transport of the vast majority of drug molecules is prevented by tight junctions formed through occludins and claudins, by drug efflux pumps, as well as by dense pericyte and astrocyte coverage 1-3. Central nervous system (CNS) disorders such as Alzheimer's disease, Parkinson's disease and glioblastoma do not - or only in advanced stages - affect the integrity of the BBB, making them very difficult to curatively treat with systemic drug therapies 2-4.

To improve drug transport to and into the brain, various strategies are being explored, including pharmacological and physical means to enhance translocation across the BBB 5-7. Pharmacological means can encompass the co-application of drugs together with osmotically active agents, such as mannitol, as well as the development of transferrin receptor-targeted drugs and drug delivery systems (DDS), which can mediate transcellular transport 5, 7. These two approaches are spatially difficult to control and consequently result in unintentional drug delivery to healthy brain areas, thereby potentially causing severe side effects 8.

Sonopermeation is based on the combination of ultrasound (US) and microbubbles (MB). It is an emerging physical procedure enabling transient BBB opening and it allows for spatially controlled (when using focused US) drug delivery to and into the brain 3, 9, 10. MB are 1-10 µm-sized, air-filled vesicles which are widely used in the clinic as US contrast agents 11. MB also hold therapeutic potential, both pharmacologically (upon drug loading into the MB shell and US-mediated local drug release) and physically (upon US-mediated MB oscillation to induce vascular permeability) 12-14. Regarding physical treatment, the application of US induces MB oscillation which results in a number of biophysical phenomena, including push/pull, microstreaming, acoustic radiation force, shock wave, jet formation and free radical formation effects 15. These effects occurring in close proximity to vascular endothelium have been assumed to be able to decompress collapsed tumor vessels in pancreatic cancer patients (which are characterized by dense stroma and inefficient blood flow), thereby improving tumor perfusion and drug delivery 16. In addition, blood vessels and endothelial cells can be permeabilized and endocytosis and transcytosis can be induced in endothelial cells. The sum of these processes has recently been coined sonopermeation 15, 17, and besides being applicable to tumors, it also holds considerable potential for drug delivery across the BBB.

Combining MB with focused US enables a temporally and spatially confined control of the BBB opening. This helps drugs and DDS to reach pathological sites, while reducing the incidence and intensity of side effects by avoiding drug accumulation in healthy parts of the brain. Two pioneering clinical trials have recently been reported where the safety and feasibility of sonopermeation for the treatment of brain disorders have been documented. Carpentier et al. employed locally implanted US devices and Mainprize et al. used externally applied focused US to enhance the delivery of low-molecular-weight chemotherapeutics (doxorubicin and carboplatin) as well as nanotherapeutics (Doxil®) to glioblastoma lesions 18, 19. Both studies reported a reversible and repeatable opening of the BBB without causing obvious side effects, providing a solid basis for further preclinical and clinical studies to employ sonopermeation to enhance the efficacy of drugs and DDS in high medical need brain disorders.

To extend these pioneering efforts, it is important to identify optimal DDS properties for translocation across a sonopermeated BBB. The size of DDS roughly ranges from 3‑200 nm, as compared to 0.5-1 nm for low-molecular-weight drugs. Small molecule drugs typically present with a suboptimal biodistribution, resulting - in the case of chemotherapeutics - oftentimes in low efficacy and high toxicity. Nanocarriers can help to improve the (spatial) biodistribution of small molecule drugs, and they can furthermore act as (temporal) drug depot systems, allowing for a sustained release of drug molecules over prolonged periods of time.

Many different types of DDS have been designed and evaluated over the years, varying greatly in chemical composition (e.g. polymers, lipids, inorganic nanoparticles) and physicochemical properties (e.g. size, loading capacity, surface charge and coating). Several of these nanomedicine formulations have been combined with US and MB to enhance drug delivery across the BBB into the brain 20-25. The balance between nanocarrier size and drug loading capacities is considered to be crucial when developing DDS for the passage across the sonopermeated BBB. In this context, Shen et al. employed three differently sized liposomes and showed that they accumulated in a size-dependent manner in the brain upon US-mediated BBB opening 26. Besides studying DDS accumulation at the macro-level, it is important to also investigate nanocarrier translocation at the micro-level, i.e. in individual vessels, to better understand how deep DDS can penetrate across a sonopermeated BBB into the brain tissue.

**Figure 1:**
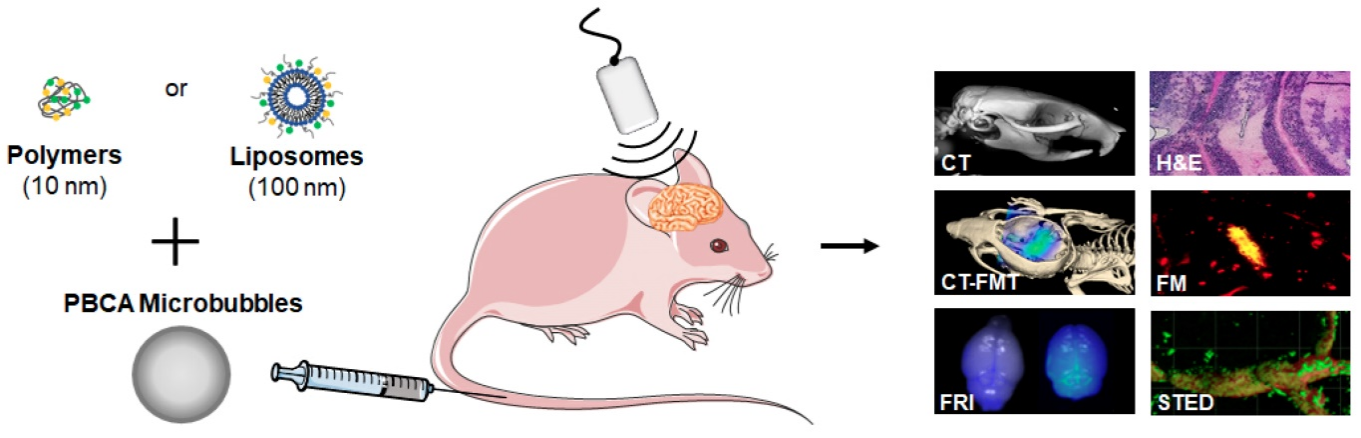
** Study setup.** Nanomedicine delivery to and into the brain upon sonopermeation-induced BBB opening was evaluated using multimodal and multiscale optical imaging. Two prototypic drug delivery systems were employed, i.e. 10 nm-sized pHPMA polymers and 100 nm-sized PEGylated liposomes. Both systems were labeled with fluorophores. Upon co-administration with poly(butylcyanoacrylate)-based (PBCA) polymeric microbubbles (MB) and the application of local transcranial ultrasound (US), the accumulation and penetration of polymers and liposomes were evaluated using several different optical imaging techniques.

Consequently, in this manuscript, we extend the above-mentioned efforts and systematically study the effect of drug-free nanocarrier size on their accumulation, extravasation and penetration after sonopermeation treatment in healthy murine brains. To this end, we employed two prototypic and clinically relevant nanocarrier systems which vary significantly in size, i.e. 10 nm‑sized polymers based on pHPMA (poly(N-(2-hydroxypropyl)methacrylamide) and 100 nm-sized PEGylated liposomes 19, 27-30. These size dimensions cover the majority of routinely used nanomedicine formulations, and they are within size range that is expected to be useful for safe and efficient drug delivery across the sonopermeated BBB. Both nanocarriers were fluorophore labeled with Alexa-488 and Alexa-750 (liposomes) or Atto 488 and Dy-750 (polymers), respectively, which enabled the employment of a large variety of optical imaging modalities, facilitating systematic analyses on accumulation, extravasation and penetration of nanocarriers into the brain 31. Figure 1 provides an overview of the applied methods and the used imaging modalities. After sonopermeation treatment, multimodal optical imaging techniques including *in vivo* hybrid Computed Tomography-Fluorescence Molecular Tomography (CT-FMT), Fluorescence Reflectance Imaging (FRI), ex vivo Fluorescence Microscopy (FM), Confocal Microscopy (CM) and Stimulated Emission Depletion (STED) nanoscopy, were employed to monitor and compare the accumulation, extravasation and penetration of 10 nm-sized pHPMA polymers and 100 nm-sized PEGylated liposomes across the BBB (Figure 1). Furthermore, potential side effects of the sonopermeation treatment, such as erythrocyte extravasation and micro-hemorrhages, were investigated histologically via hematoxylin and eosin (H&E) staining. Additionally, the opening of the BBB was verified by the assessment of permeabilized vessels, identified by means of extravasated immunoglobulin G (IgG). Our findings illustrate that sonopermeation is a secure procedure to enhance the accumulation, extravasation and penetration of 10 nm-sized pHPMA polymers to a much greater extent than 100 nm-sized PEGylated liposomes in brain tissue. Further, the employment of multimodal optical imaging techniques at a preclinical level may facilitate a deeper understanding of nanocarrier accumulation and penetration to and into the brain.

## Results and Discussion

### *In vivo* and ex vivo visualization of sonopermeation-mediated nanomedicine accumulation in brain

To study the effect of sonopermeation on nanocarrier accumulation and penetration in mouse brains, fluorophore-labeled pHPMA polymers (10 nm) and PEGylated liposomes (100 nm) were intravenously (i.v.) injected into healthy nude mice via the tail vein (Figure 1). Animals were randomly assigned to the sonopermeation group or to the control group. Sonopermeation was restricted to the brain of the mice. Transcranial US was applied using a Vevo® 2100 Imaging System (FUJIFILM, VisualSonics Inc.) for 5 min, at a frequency of 16 MHz and a peak negative pressure of 1.8 MPa, resulting in a mechanical index (MI) of 0.45. In-house prepared poly(butylcyanoacrylate) (PBCA) microbubbles were i.v. infused via the tail vein at a concentration of 1*10^7^ MB / 20 µl / minute during the full duration of US application 32.

**Figure 2:**
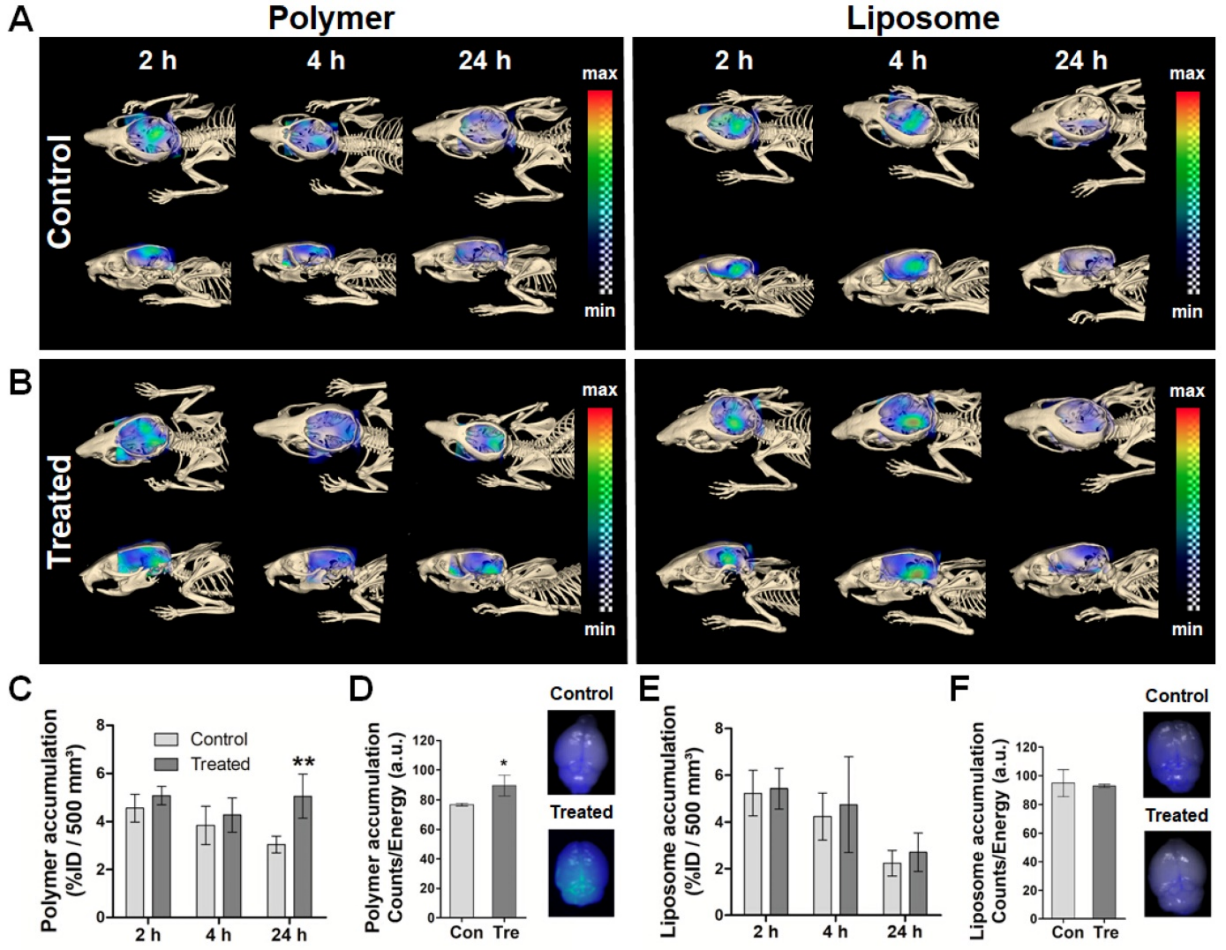
** Longitudinal assessment of sonopermeation-enhanced nanocarrier accumulation in the brain.** A,B: CT-FMT images of 10 nm polymer and 100 nm liposome accumulation in the brain of healthy mice upon sonopermeation vs. control treatment. Cranial bone is adapted to allow for a view inside the skull. C,E: Quantification of the CT-FMT data sets shows that polymer accumulation is significantly increased at 24 h after sonopermeation (C), which is not the case for liposomes (E). D,F: Ex vivo FRI analyses of excised brains confirm that sonopermeation-enhanced nanocarrier translocation across the BBB is more efficient for 10 nm polymers than for 100 nm liposomes. **=p<0.01.

Sonopermeation-mediated nanocarrier translocation across the BBB was monitored using hybrid CT-FMT at 2, 4 and 24 h post DDS injection (p.i.). The transversal and sagittal CT-FMT images in Figure 2A-B indicate that the effect of sonopermeation was stronger for 10 nm-sized polymers than for 100 nm-sized liposomes, particularly after 24 h. In line with this, quantification of the amounts of DDS present in the brain at 24 h p.i. showed a significantly higher accumulation of polymers in sonopermeated brains as compared to control brains (5.1 ± 0.9 % vs. 3.1 ± 0.3 % of the injected dose (%ID) per 500 mm³ of brain tissue; p<0.01; Figure 2C and 2E). In the case of liposomes, only a slight increase in accumulation was observed in the US-treated group as compared to the control group at 24 h p.i. (2.7 ± 0.8 % vs. 2.2 ± 0.6 %; p>0.05; Figure 2A, 2B and 2E). The relatively high ID% values for non-treated control animals can be explained by the prolonged blood half-life of pHPMA polymers and PEGylated liposomes. After *in vivo* CT-FMT, mice were euthanized and brain tissues were harvested. The semi-quantitative ex vivo FRI analysis in Figure 2D and 2F confirms significantly enhanced accumulation for polymers upon sonopermeation.

Taken together, these findings are in good agreement with previously published papers and the general notion that sonopermeation can help to improve the delivery of drugs and DDS across the BBB 9, 10, 33, 34. We extend this knowledge by showing that sonopermeation enhances the accumulation of 10 nm polymers to a greater extent than that of 100 nm liposomes (+65% enhancement for polymers; +35% enhancement for liposomes; total brain accumulation: 5.1 ± 0.9 %ID for polymers vs. 2.7 ± 0.8 %ID for liposomes). These notions confirm our hypothesis that sonopermeation-mediated BBB opening enables smaller sized nanocarriers to pass through the endothelium and enter the brain parenchyma more efficiently compared to larger sized nanocarriers.

### Safety assessment and efficacy validation of sonopermeation-mediated BBB opening

**Figure 3:**
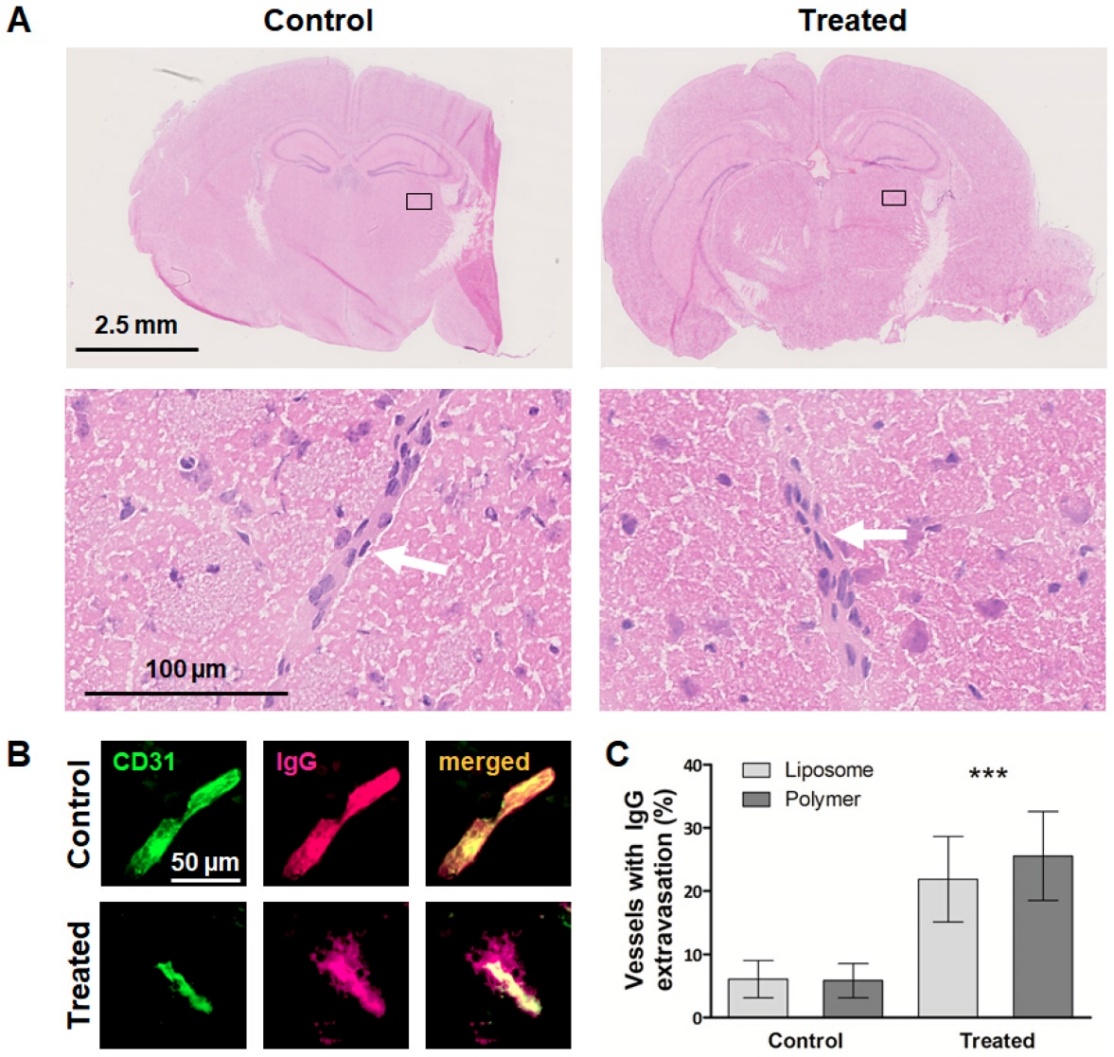
** Safety assessment and confirmation of sonopermeation-mediated BBB opening.** A: Representative H&E stainings of non-treated and US-treated mice. No tissue or vessel damage could be observed in both groups and a clear delineation of the vessel border was possible (white arrow). B: Extravasated IgG was used as a biomarker for the evaluation of BBB opening via ex vivo immunohistological stainings of vessels (CD31, green) and endogenous IgG (pink). IgG signal overlapped with the vessel signal while in sonopermeated animals, extravasated IgG was detectable. Scale bar: 50 µm. C: Image evaluation revealed that sonopermeated animals presented with a significantly higher amount of vessels with extravasated IgG compared to control animals. ***=p<0.0001.

We subsequently assessed the safety of sonopermeation-based BBB opening, using H&E stained, cryosectioned brain tissues to assess the integrity of gray matter and small vessels 35. In parallel, we visualized and quantified endogenous IgG extravasation in these sections, to validate the BBB opening, and to confirm that the extent of BBB permeation was similar in the polymer and liposome study setup. In the case of BBB sonopermeation experiments, H&E staining is routinely used to detect erythrocyte extravasation, micro-hemorrhages, edema or necrosis, which are all signs of brain damage 36, 37. As demonstrated in H&E images in Figure 3A, in our experimental setup, we did not observe obvious signs of erythrocyte extravasation, micro-hemorrhages, edema and necrosis, and staining patterns looked identical for sonopermeated and control brain sections. These findings confirm our macroscopic observations of animal behavior during and after BBB sonopermeation, in which we could not detect any differences between treated and untreated animals.

In parallel with the safety assessment experiments, we validated the efficiency of the sonopermeation protocol in the treated vs. untreated polymer and liposome groups. This was done via antibody-based staining of the extravasation of endogenous immunoglobulin G (IgG), which can serve as a marker for BBB opening because it is unable to cross the intact BBB 38-40. Vessels were counterstained using CD31, and the percentage of vessels presenting with extravasated IgG was visualized and quantified. As shown in Figure 3B-C and Supplementary Figure 1, the percentage of vessels positive for IgG extravasation was significantly higher upon sonopermeation than upon sham treatment (23.7 ± 6.5 % vs 6.0 ± 2.7 %; p<0.0001), and the extent of BBB opening was similar in the polymer and liposome cohort (25.5 ± 7.0 % vs 21.9 ± 6.7 %; p>0.05). Together, these findings demonstrate that the employed sonopermeation protocol was well tolerated and enabled efficient and consistent BBB opening.

### Fluorescence microscopy analysis of nanomedicine extravasation and penetration

**Figure 4:**
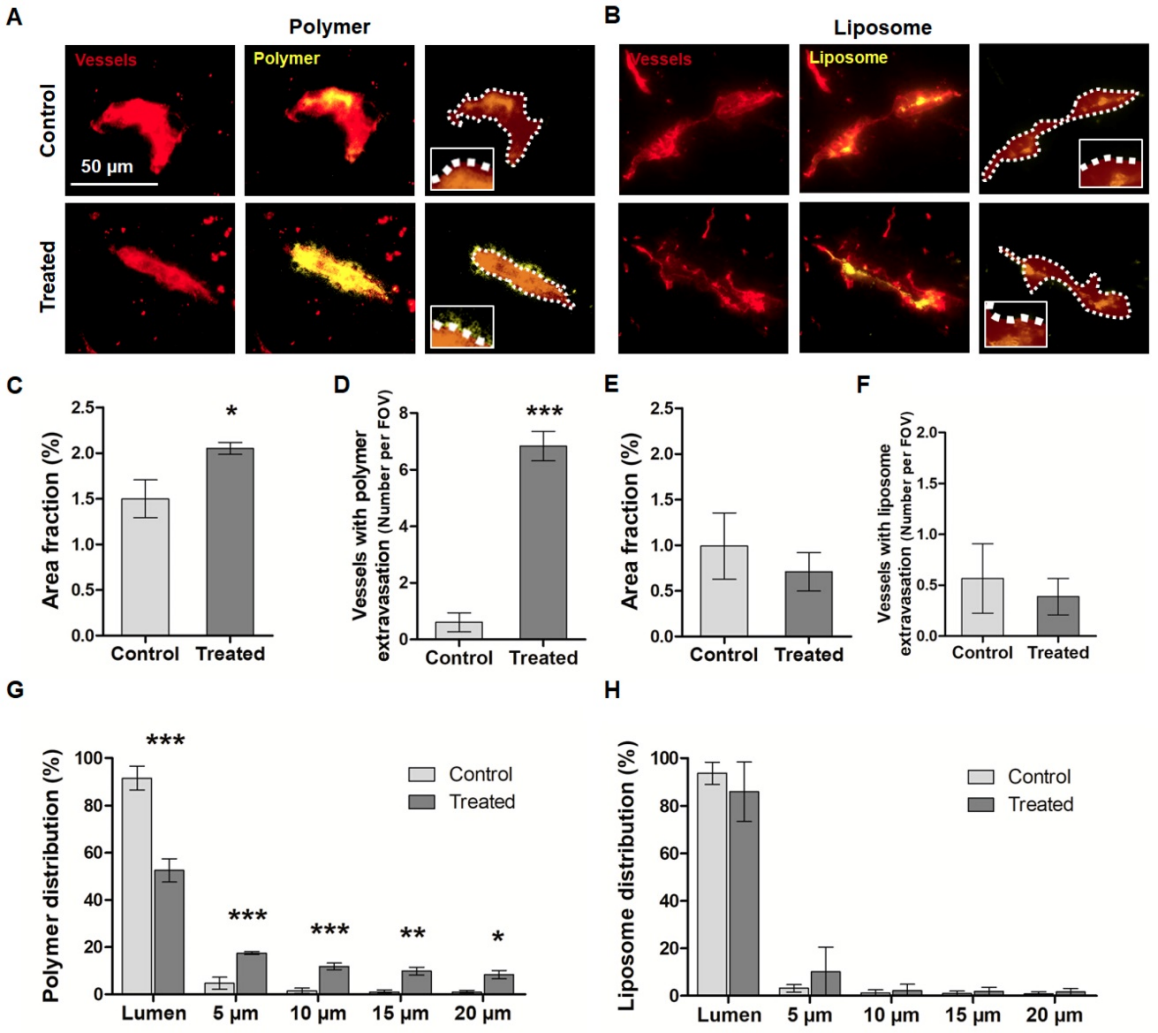
** Fluorescence microscopy analysis of nanocarrier accumulation, extravasation and penetration in the brain upon sonopermeation.** A,B: Fluorescent microscopy images of brain sections from control and treated animals (vessels in red, nanocarriers in yellow). Scale bar: 50 µm. The dashed white lines indicate the vessel boundaries which were filled with a transparent red color to allow a better visualization of the yellow DDS signal. C-F: Sonopermeation significantly enhanced the area fraction for 10 nm polymers (C) and the percentage of vessels positive for polymer extravasation (D), while it did not affect both parameters for liposomes (E-F) G-H: Distribution analyses of polymers and liposomes revealed efficient and deep penetration upon sonopermeation for polymers (G), but not for liposomes (H). ***=p<0.001, **=p<0.01, *=p<0.05.

In the next set of experiments, we used different types of fluorescence microscopy to evaluate the extent to which nanocarriers penetrate into the brain upon sonopermeation. To this end, fluorescence microscopy images were obtained, and the area fraction of polymers and liposomes with and without sonopermeation was determined (Figure 4). We also analyzed the percentage of vessels positive for polymer and liposome extravasation, as well as the penetration depth of both nanocarriers away from the vessels into the brain parenchyma. As shown in Figure 4A, we could clearly detect 10 nm polymer extravasation upon sonopermeation using standard fluorescence microscopy. Figure 4B shows that this was not the case for 100 nm liposomes. The area fraction positive for polymers significantly increased upon treatment, from 1.5 ± 0.2 % to 2.1 ± 0.1 % (p<0.05; Figure 3C), and also the percentage of vessels positive for polymer extravasation increased (0.6 ± 0.3 vs. 6.8 ± 0.5 vessels/field-of-view; p<0.001; Figure 4D). In contrast, in the case of liposomes, the area fraction and the percentage of vessels which were positive for liposome extravasation did not change upon sonopermeation (Figure 4E-F). These findings are in line with the above presented *in vivo* CT-FMT and ex vivo FRI data, and they support the notion that smaller-sized nanocarriers benefit more from sonopermeation.

We furthermore visualized and quantified nanocarrier penetration into different compartments beyond the vessel lumen. For this purpose, a Definiens-based script was prepared to automatedly detect the rhodamine-lectin-stained vasculature, which was then gradually expanded in two dimensions via drawing consecutive concentric rings of 5 µm. The fluorescent signal coming from the polymers and liposomes were quantified within the blood vessel, and in the compartments 5 µm, 10 µm, 15 µm and 20 µm away from the vessel lumen.

As shown in Figure 4G, in case of the polymeric DDS, the percentage confined to the vasculature decreased from 92 ± 5 % to 53 ± 5 % upon US treatment (p<0.001). In addition, sonopermeation significantly enhanced the penetration of polymers into deep compartments of the brain, with 18 ± 1 %, 12 ± 1 %, 10 ± 2 % and 8 ± 2 % of the polymers per field-of-view present in the 5 µm, 10 µm, 15 µm and 20 µm compartment, respectively. In untreated control brains, only 5 ± 3 %, 2 ± 1 %, 1 ± 1 % and 1 ± 1 % of the polymer signal could be localized per compartment, respectively (p<0.05 for all relevant comparisons). This result is in line with previously published findings on a similarly sized model nanocarrier, i.e. 70 kDa FITC dextran, which also accumulated and penetrated much better into the brain upon sonopermeation 34. When looking at liposome penetration, there was a decrease in the percentage presented within the vessel lumen upon sonopermeation, but this was not significant compared to control (94 ± 5 % vs. 86 ± 13 %, respectively; p>0.05). In the deeper compartments, 2-3-fold higher levels were observed, but these differences never reached significance: 10 ± 10 %, 2 ± 3 %, 2 ± 2 % and 2 ± 1 % of the liposomes were present per field-of-view in the 5 µm, 10 µm, 15 µm and 20 µm compartment upon sonopermeation, respectively, as compared to 3 ± 2 %, 1 ± 1 %, 1 ± 1 % and 1 ± 1 % for control (p>0.05 for all comparisons). These findings demonstrate that sonopermeation promotes brain penetration more efficiently for 10 nm polymers than for 100 nm liposomes. This notion is in line with our hypothesis, as well as with previous reports looking at the accumulation and penetration of differently sized nanocarriers in other tissue types, particularly tumors 41-43. Together, these findings substantiate the general dogma that nanocarriers should be large enough to avoid renal clearance, but at the same also small enough to allow for proper tissue penetration.

### Confocal microscopy and STED nanoscopy analysis of nanomedicine penetration upon sonopermeation

**Figure 5:**
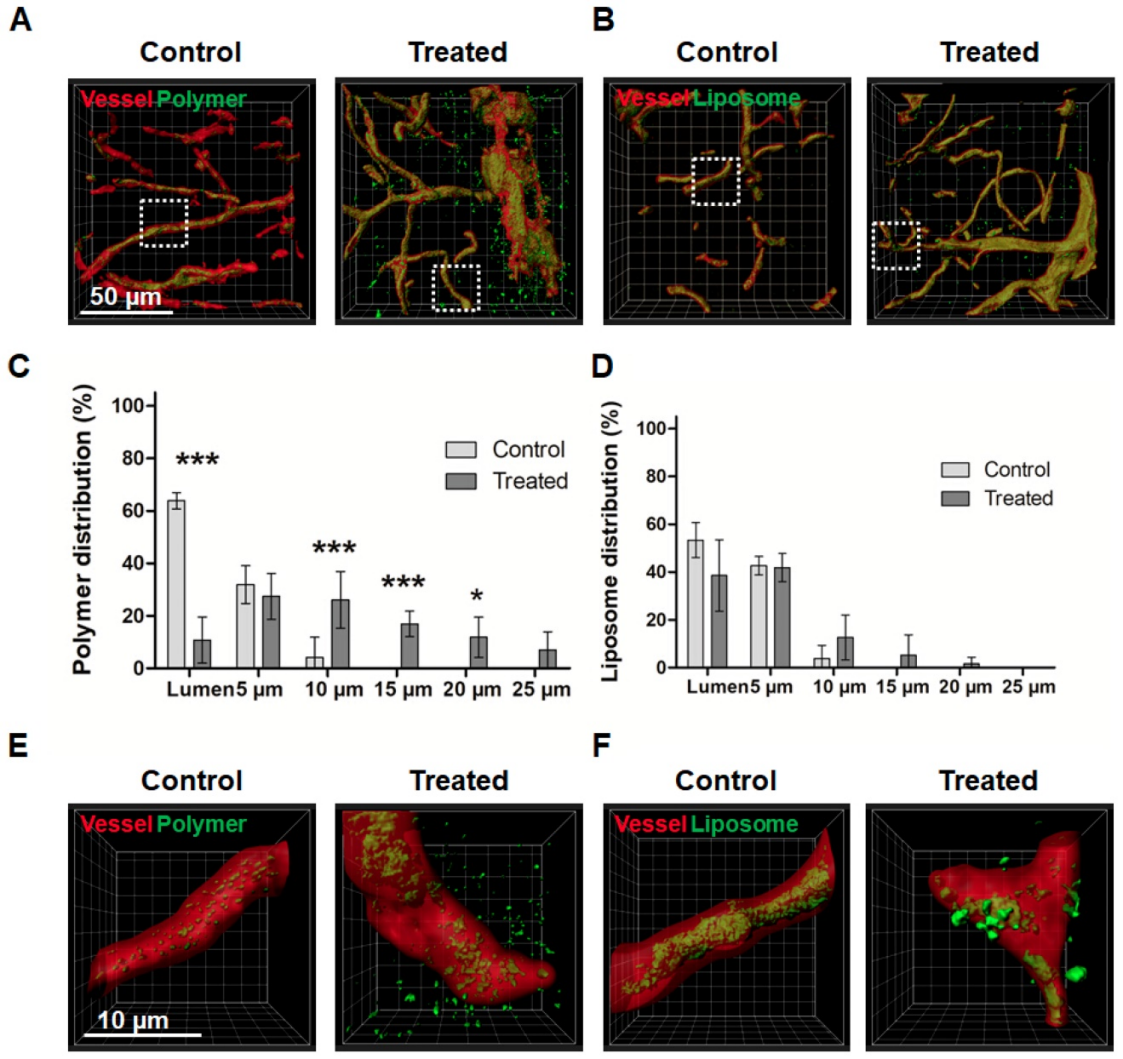
** Confocal microscopy and STED nanocopy analysis of nanocarrier penetration into the brain upon sonopermeation.** A-B: Confocal microscopy (CM) images of polymer (A) and liposome (B) penetration upon sonopermeation, asssesed in ~25-40 µm-thick brain sections. Vessels are depicted in red (rhodamine-lectin), nanocarriers in green. C-D: By rendering vessel surfaces and extending them with 3D concentric rings in the CM images, the penetration and distribution of nanocarriers in the brain was evaluated. Polymers were found to penetrate more efficiently into the brain tissue than liposomes. E-F: Stimulated emission depletion microscopy visualized deeper penetration and better distribution of polymeric nanocarriers as compared to liposomal nanocarriers. ***=P<0.001, *=p<0.05.

Following the fluorescence microscopy analyses, the penetration of 10 nm polymers and 100 nm liposomes into the brain was also studied via confocal microscopy (CM) and stimulated emission depletion (STED) nanoscopy. These two modalities allow for 3D imaging of thicker tissue sections, and they provide a much higher spatial resolution compared to standard fluorescence microscopy. CM imaging could clearly visualize the extravasation and penetration of polymers upon sonopermeation (Figure 5A). For liposomes, fluorescent signals were detected outside of blood vessels upon treatment, but much more sparsely as compared to polymers, and also less homogenous distributed over the evaluated volume of brain tissue (Figure 5B).

A MATLAB-based script was applied for the 3D quantification of the DDS signal in relation to the vessel lumen and for the stepwise analysis of nanocarrier penetration beyond the BBB into deeper brain parenchyma compartments 44. In the case of polymers, sonopermeation promoted delivery beyond the vessel lumen by more than 600% (63.9 ± 3.1 % in the lumina compared for control vs. 10.7 ± 8.8 % in the treatment group; p<0.001; Figure 5C), in the case of liposomes, the enhancement was only approximately 25% (53.3 ± 7.4 % in the lumina compared for control vs. 38.5 ± 14.9 % in the treatment group; Figure 5D). Both for polymers and for liposomes, penetration into deep brain compartments was substantially enhanced upon sonopermeation. For 100 nm liposomal DDS, 1.6 ± 2.8 % of the total dose that accumulated in the brain managed to penetrate 15-20 µm (Figure 5D). For 10 nm polymeric DDS, 11.8 ± 7.7 % of the dose that accumulated in the brain penetrated 15-20 µm deep, and 7.0 ± 7.0 % even penetrated 20-25 µm (Figure 5D). Without sonopermeation, no polymers and liposomes were detected beyond 10 µm of the rendered vessel lumen surfaces (Figures 5C-D).

These CM observations extend the abovementioned results acquired by FM (Figure 4), as in this case, also extravasated liposomes could be visualized upon sonopermeation (Figure 5B). This discrepancy can be explained by the fact that the spatial resolution of CM is higher than that of FM. The notion that liposomes indeed manage to extravasate across the BBB upon sonopermeation was validated via STED nanoscopy (Figures 5E-F). STED nanoscopy has emerged as a powerful and high-resolution imaging technique which can overcome the diffraction limitations of optical microscopy 45. It has a spatial resolution in the range of a single liposome (i.e. 100 nm), and it consequently is a highly suitable and sensitive tool for studying nanomedicine penetration and distribution in target tissues. In our study, the resolution of the images was further improved by deconvolution (see Figure S1). The scanning locations for STED nanoscopy were chosen based on confocal z-stack scans (dotted squares in Figure 5A-B). The processed and deconvolved STED images in Figures 5E-F nicely captured the penetration and distribution of polymers and liposomes upon sonopermeation, visualizing the findings obtained using FM and CM.

Taking everything together, we here demonstrate that sonopermeation enhances the brain accumulation and penetration of 10 nm-sized pHPMA polymers to a greater extent than of 100 nm-sized PEGylated liposomes. As the closing of the BBB upon US- and MB-mediated opening is a continuous and size-dependent process 46, these results can be explained by the fact that the time window for BBB crossing and thus for accumulation is longer for 10 nm-sized DDS than for 100 nm-sized DDS. In addition, when aiming to ensure safe BBB opening, the median pore sizes likely cannot be large, thus favoring smaller formulations. Furthermore, upon overcoming the initial vascular barrier, smaller-sized nanocarriers likely diffuse more efficiently and thus deeper into the brain than larger-sized nanocarriers. As compared to small molecule drugs, however, which may extravasate and penetrate even deeper than 10 nm polymeric nanocarriers, a key advantage of using DDS is that they enable temporal control over drug activity, releasing e.g. 5% of the active ingredient per day. This contributes to increasing the therapeutic gain upon combining nanomedicine formulations with sonopermeation, since in clinically relevant scenarios, sonopermeation can likely only be applied once every couple of weeks, and thus DDS are needed that upon initial deposition across the BBB can controllably release their contents at the target site over prolonged periods of time. In this context, a critical issue to consider is drug loading capacity. Polymeric nanomedicines with a size of 5-20 nm can typically only carry 2-5 drug molecules, whereas 100 nm-sized liposomes can carry 1000's of drug molecules 47, 48. Regarding targeted drug therapy upon BBB sonopermeation, the results presented here therefore do not necessarily mean that 10 nm-sized polymeric DDS will perform better in terms of therapeutic response than 100 nm-sized liposomes. Instead, they are intended to serve as a starting point and they provide a proper framework for follow-up experiments involving the combined use of sonopermeation and DDS for improving the treatment of high medical need CNS disorders.

## Conclusion

We here show that US- and MB-based sonopermeation can be employed to open the BBB in a safe and efficient manner, enabling the extravasation of 10 and 100 nm-sized nanomedicine formulations in the brains of healthy mice. By employing multimodal and multiscale optical imaging techniques, we show that smaller-sized DDS accumulate better and penetrate deeper in mouse brains than larger-sized DDS. These findings exemplify the potential of sonopermeation for nanomedicine delivery across the BBB, and they showcase the value of multimodal and multiscale optical imaging for systematically studying drug delivery processes at the preclinical level. On the long run, such systematic head-to-head comparisons of (differently sized) DDS will contribute to the identification of nanomedicine formulations that have optimal properties with respect to extravasation and penetration, as well as drug loading capacity and drug release kinetics, for efficient treatment of high medical need CNS pathologies, such as brain tumors and neurodegenerative disorders.

## Material and Methods

### Synthesis and characterization of microbubbles, liposomes and polymers

PBCA-based hard-shell MB were synthesized as described previously 32. Briefly, the n-butyl cyanoacrylate monomer was added to an aqueous solution containing 1% (w/v) tritonX-100 at pH 2.5 in a drop-wise manner. The solution was continuously mixed using an ultra-turrax (IKA-Werke) until the monomer was completely added, followed by stirring at 10,000 rpm for 60 min. Several washing and purification steps resulted in PBCA-MB with a size of 1.5-3 µm.

Liposomes were prepared as in 49. In short, double-labeling was achieved by adding Alexa-488 and Alexa-750 labeled PEG-PE micelles via a post-insertion method into pre-prepared liposomes. The PEG-PE micelles based on PEG(2000)-DSPE-NH_2_ and PEG(2000)-DSPE were obtained upon covalently linking the NHS esters of the Alexa-488 and Alexa-750 dyes to the lipids. The double-labeled micelles were mixed with liposomes and the solution was heated for 5 min to 60°C followed by 10 min at room temperature. Heating was repeated 3 times. Finally, liposomes with a diameter of 100 nm and PDI<0.1 were obtained upon lipid film hydration and extrusion. The particle size was determined by dynamic light scattering on an ALV CGS-3 system (Malvern Instruments) and the zeta potential was measured by using a Zetasizer Nano Z (Malvern Instruments) 50.

Polymers were synthesized as in 51-53. Radical copolymerization of *N*-(2-hydroxypropyl) methacrylamide (HPMA; 85 mol %) and 3-(*N*-methacryloyl glycylglycyl)thiazolidine-2-thione (Ma-GG-TT; 15 mol %) in DMSO at 50°C for 6 h yielded the copolymer precursor poly(HPMA-co-Ma-GG-TT). The fluorophores Atto 488-NH_2_ and Dy750-NH_2_ were added to the solution of the polymer precursor (10% w/w) in *N,N*-dimethylacetamide and *N,N'*-diisopropylethylamine (equimolar amount related to the fluorophores) was added. The remaining reactive TT groups of the polymer were aminolyzed with 1-aminopropan-2-ol and precipitated with diethylether after 30 min. The crude product was purified using gel filtration on PD-10 desalting columns containing Sephadex G-25 resins in water. Size-exclusion chromatography equipped with refractive index and multi-angle light scattering detectors (Wyatt Technology) revealed a hydrodynamic radius (Rh) of 4.1 nm and a polydispersity index of 1.7 for the 67 kDa pHPMA polymer. In physiologically relevant solutions, e.g. in blood plasma, the size of these coiled-coil polymers was determined to be 10-20 nm via fluorescence correlation spectroscopy 42. Dye contents were 2.1 %w/w for Atto488 and 1.6 %w/w for Dy750 as assessed by UV/Vis spectrophotometry.

### *In vivo* experiments

A governmental review committee on animal care approved all animal experiments. Sixteen CD-1 nude mice were obtained from Charles River Laboratories and used at an age of 8 weeks. Mice were split into 4 groups: 5 animals received an i.v. injection of liposomes with sonopermeation or liposomes without sonopermeation, 3 animals received an i.v. injection of polymers with sonopermeation or polymers without sonopermeation. All *in vivo* experiments were conducted under continuous inhalation anesthesia using 2 %v/v isoflurane. One hour before sonopermeation, animals received an i.v. injection of nanocarriers adjusted to a dose containing 4 nmol of dye. For sonopermeation, 100 µl containing 5 x 10^7^ PBCA-MB were infused over 5 min, overlapping with US application. A CT-FMT pre-scan was acquired 15 min prior to sonopermeation as well as at 2, 4 and 24 h post US treatment. Functional vessels were visualized via the i.v. injection of rhodamine‑lectin. Mice were sacrificed using a lethal dosage of isoflurane followed by an exsanguination via the opening of the vena cava. The brains were resected, their fluorescence was imaged via the FMT 2500 LX (Perkin Elmer) acquiring FRI scans followed by embedding of the brains in TissueTek ® (Sakura Finetek) and storage at -80°C.

### Sonopermeation protocol

The used US device was a Vevo® 2100 Imaging System (FUJIFILM VisualSonics Inc.) attached with a MS250 transducer. Mice were placed below the fixed transducer on a custom-made heating bed which was constantly and linearly moved during the US treatment to guarantee a whole-brain US application. Power Doppler US was used with 50% power, a frequency of 16 MHz and a peak negative pressure of ~1.8 MPa leading to a mechanical index of 0.45 over 5 min. As mentioned above, 5 x 10^7^ PBCA-MB were infused in parallel to the US treatment to guarantee a continuous sonopermeation effect during the treatment with an expected focal plane roughly 3 mm below the skin.

### CT-FMT imaging

The accumulation of polymers and liposomes with and without sonopermeation was non-invasively and longitudinally monitored via CT-FMT imaging, employing a Micro-CT Tomoscope 30s Duo (CT-Imaging) and a FMT 2500 LX (Perkin Elmer). For CT measurements, 2 sub-scans of the head region were acquired with the tubes operating at 65 KV and a current of 0.5 mA, capturing 720 projections with 1032x1032 pixels in a full rotation over a duration of 90 s. Image reconstruction was done as described in 54, 55. For FMT images, the head of the mouse was scanned using approximately 50 scan points distributed in a 3x3 spaced mm grid. The 750 nm channel was used for acquisition and each animal was scanned in up- and the downward position. Images were reconstructed, overlayed and analyzed using the IMALYTICS Preclinical Software 54, 56. The CT- based segmentation of the brain was used to determine the FMT-detected amount of fluorescence (and nanocarrier concentration) in the brain, eventually resulting in values expressed as %ID/500 mm³.

### Microscopy analysis

Histological analysis of nanocarrier accumulation and distribution was done using an Axio Imager M2 fluorescence microscopy (Carl Zeiss). Brain tissue was cut in 8 µm slices (3 slices per brain) using a cryotome (Cryostat CM3050 S, Leica) and images were acquired (4 images per slice) using the DsRed (for vessels), GFP and/or Cy7 (for polymers and liposomes) channels, using a 20x objective. For analysis of the fluorescence microscopy images, the open source FiJi software was used 57.

Confocal and STED microscopy analyses were performed on a Leica TCS SP8 microscope (Leica). Fluorescently labeled pHPMA polymers (Atto 488) and liposomes (Alexa-488) as well as rhodamine-lectin (functional vessels) were used to visualize and analyze the distribution or extravasation of nanocarriers in brain tissue. Slices of ~25-40 µm were embedded in Mowiol^®^ (Carl Roth) and deposited on a high precision cover glass (170 µm, No. 1.5H) from Marienfeld. A 93X/1.30 glycerol objective with glycerol immersion liquid type G (refractive index: 1.45) and a white light laser source were used in the measurements. The Atto 488- as well as the Alexa-488-labeled nanocarriers were excited at a wavelength *λ* = 498 nm and the resulting emission was detected at *λ* = 509-542 nm, while the rhodamine-lectin-labeled vessels were excited with *λ* = 550 nm and the corresponding emission was detected at *λ* = 560-650 nm. 3D images were generated by stack-scanning measurements in the Z direction with a step size of 500 nm. To perform STED microscopy analyses, Atto 488- and Alexa-488-labeled nanocarriers were sequentially irradiated with a 592 nm STED laser, while the rhodamine-labelled vessels were irradiated with a 775 nm STED laser. Raw images were processed with Huygens Professional (Scientific Volume Imaging) and Imaris Software (Version 7.4; Bitplane AG).

Extravasated endogenous mouse IgG was stained with horse anti-mouse IgG HRP-labeled (Vector Laboratories) via the TSA dye Cy5.5 (Perkin Elmer). For H&E staining, cryosections with a thickness of 4 µm were processed in an automated staining system (TissueTek Prisma; Sakure Finetek). Images were acquired using a whole-slide scanner (NanoZoomer 2.0 HT; Hamamatsu) and evaluated by a pathologist in a blinded manner.

Nanocarrier distribution was evaluated via the Definiens Developer XD Software (Definiens AG) in case of the fluorescence microscopy images. In case of the confocal and STED images, the Imaris Software was used (Version 7.4; Bitplane AG). The nanocarrier micro-distribution was processed and analyzed as described previously 44 using a modified version of the MATLAB-based “Dilate Surface” XTension in Imaris.

### Statistical analysis

Statistical analysis was performed using GraphPad Prism 5. All results are shown as average ± standard deviation. The results of FRI, IgG extravasation and FM (AF and counting) were analyzed via Student's t-test as only two groups were compared. CT-FMT, FM (distribution) and CM analysis were performed by using two-way ANOVA, corrected for multiple comparisons (Bonferroni). P-values less than 0.05 were considered statistically significant (*p<0.05, **p<0.01, ***p<0.001).

## Supplementary Material

Supplementary figures.
