# Supplementary material for: Multimodal and multiscale optical imaging of nanomedicine delivery across the blood-brain barrier upon sonopermeation
Source: Theranostics. 2020 Jan 12;10(4):1948–59. doi: 10.7150/thno.41161 (PMC6993230; doi:10.7150/thno.41161)
Supplement: Supplementary file 1 — Supplementary figures. [file thnov10p1948s1.pdf]

- Supplementary Material -

Multimodal and multiscale optical imaging of nanomedicine delivery across the blood-brain barrier upon sonopermeation

Jan-Niklas May[1,#], Susanne K. Golombek[1,#], Maike Baues[1], Anshuman Dasgupta[1], Natascha Drude[1], Anne Rix[1], Dirk Rommel[2], Saskia von Stillfried[3], Lia Appold[1], Robert Pola[4], Michal Pechar[4], Louis van Bloois[5], Gert Storm[5], Alexander J. C. Kuehne[2,6], Felix Gremse[1], Benjamin Theek[1,7], Fabian Kiessling[1,7], Twan Lammers[1,5,8]

1. Institute for Experimental Molecular Imaging (ExMI), University Clinic and Helmholtz Institute for Biomedical Engineering, RWTH Aachen University, Aachen, Germany
2. DWI – Leibniz Institute for Interactive Materials, RWTH Aachen University, Aachen, Germany
3. Institute of Pathology, University Clinic RWTH Aachen, Aachen, Germany
4. Czech Academy of Sciences, Institute of Macromolecular Chemistry, Prague, Czech Republic
5. Department of Pharmaceutics, Utrecht Institute for Pharmaceutical Sciences, Utrecht University, Utrecht, The Netherlands
6. Institute of Organic and Macromolecular Chemistry, Ulm University, Ulm, Germany
7. Fraunhofer MEVIS, Institute for Medical Image Computing, Aachen, Germany
8. Department of Targeted Therapeutics, University of Twente, Enschede, The Netherlands

Supplementary Figure 1:

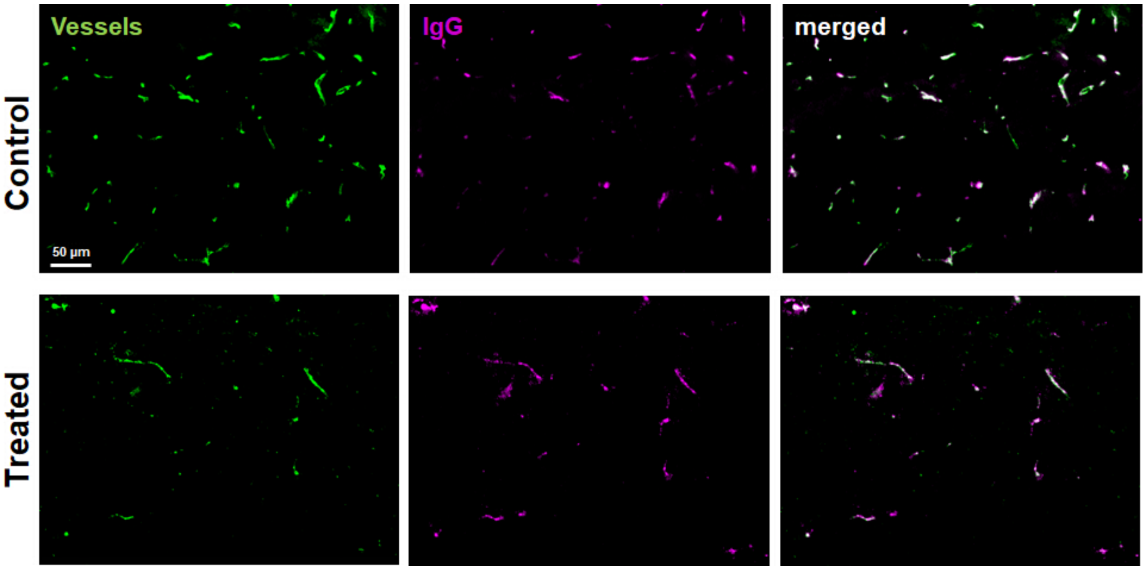

Supplementary Figure 1: Fluorescence microscopy of IgG extravasation. Immunofluorescence staining of endogenous IgG (in purple) and blood vessels (CD31 staining; in green) in the brain showed extravasated IgG only in case of sonopermeation treatment. Images at higher resolution are provided in Figure 3.

Supplementary Figure 2:

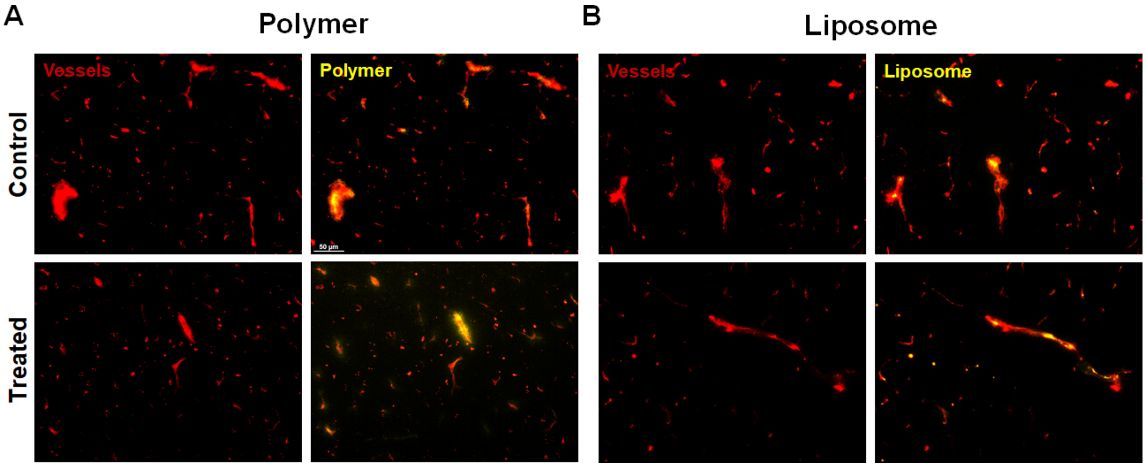

Supplementary Figure 2: Fluorescence microscopy of polymer and liposome extravasation. Analysis of nanocarrier (in yellow; fluorophore-labeled) extravasation and penetration out of brain blood vessels (in red; rhodamine-lectin staining) upon sonopermeation treatment. Higher resolution images are provided in Figure 4.

Supplementary Figure 3:

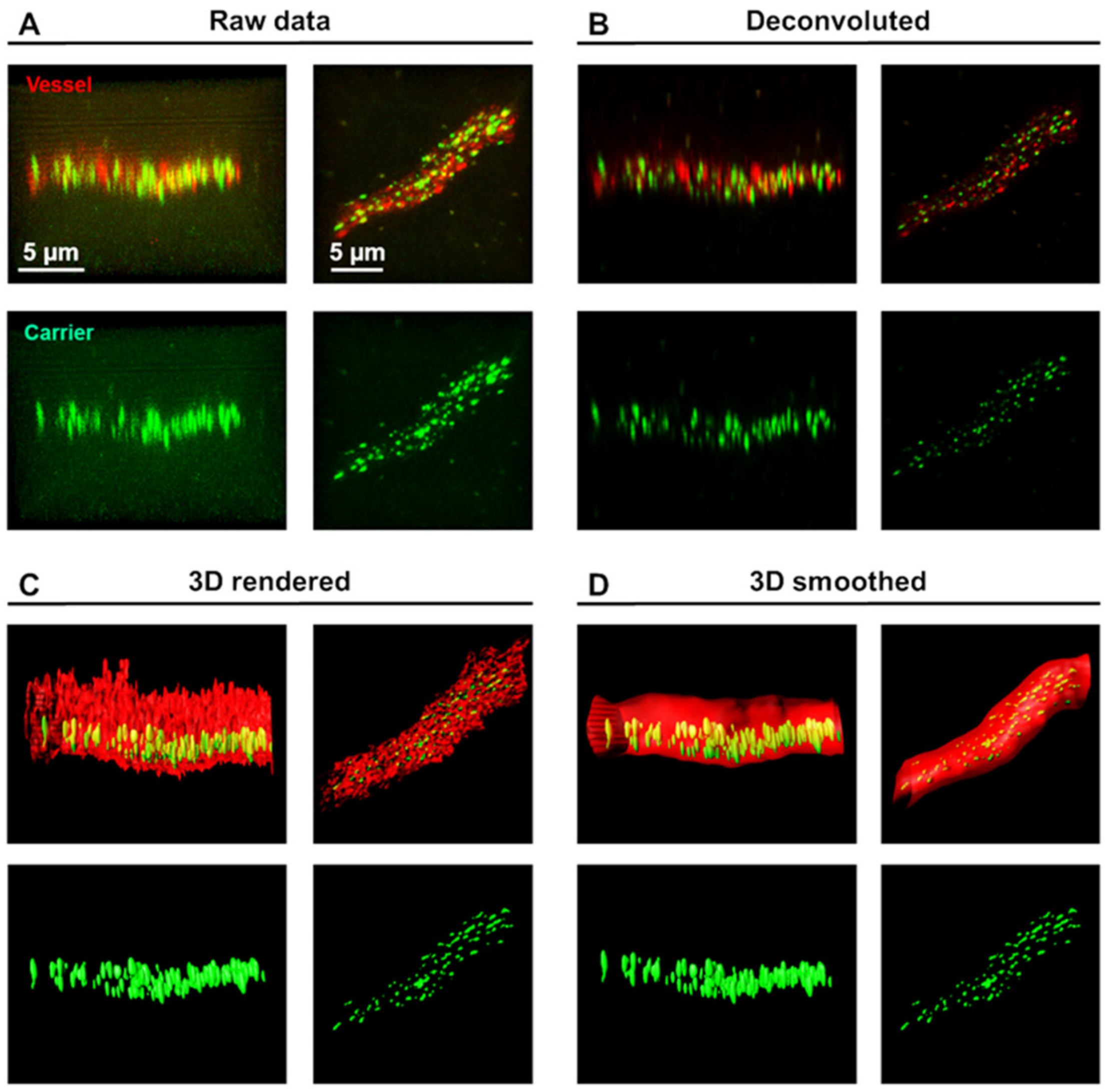

Supplementary Figure 3: STED image processing and 3D rendering. STED images of a vessels in the brain (stained using rhodamine-lectin; in red) and nanomedicine formulations (in green), at different stages of image processing. A: Raw data with background and shifted signal. B: Deconvolved images with background reduction and correction for potential signal shifts, automatically performed by the Huygens Professional software (Scientific Volume Imaging). C: 3D-rendered images based on the gained signals. D: 3D-smoothened final images, with realistic vessel shapes and surfaces.
